# Seqping: gene prediction pipeline for plant genomes using self-training gene models and transcriptomic data

**DOI:** 10.1186/s12859-016-1426-6

**Published:** 2017-01-27

**Authors:** Kuang-Lim Chan, Rozana Rosli, Tatiana V. Tatarinova, Michael Hogan, Mohd Firdaus-Raih, Eng-Ti Leslie Low

**Affiliations:** 1 Advanced Biotechnology and Breeding Center, Malaysian Palm Oil Board, 6 Persiaran Institusi, Bandar Baru Bangi, 43000 Kajang, Selangor Malaysia; 2 Center for Personalized Medicine and Spatial Sciences Institute, University of Southern California, Los Angeles, CA USA; 3 Orion Genomics, 4041 Forest Park Avenue, St. Louis, MO 63108 USA; 4 Faculty of Science and Technology, Universiti Kebangsaan Malaysia, 43600 Bangi, Selangor Malaysia

**Keywords:** Gene prediction, Gene model, Species specific HMM

## Abstract

**Background:**

Gene prediction is one of the most important steps in the genome annotation process. A large number of software tools and pipelines developed by various computing techniques are available for gene prediction. However, these systems have yet to accurately predict all or even most of the protein-coding regions. Furthermore, none of the currently available gene-finders has a universal Hidden Markov Model (HMM) that can perform gene prediction for all organisms equally well in an automatic fashion.

**Results:**

We present an automated gene prediction pipeline, Seqping that uses self-training HMM models and transcriptomic data. The pipeline processes the genome and transcriptome sequences of the target species using GlimmerHMM, SNAP, and AUGUSTUS pipelines, followed by MAKER2 program to combine predictions from the three tools in association with the transcriptomic evidence. Seqping generates species-specific HMMs that are able to offer unbiased gene predictions. The pipeline was evaluated using the *Oryza sativa* and *Arabidopsis thaliana* genomes. Benchmarking Universal Single-Copy Orthologs (BUSCO) analysis showed that the pipeline was able to identify at least 95% of BUSCO’s plantae dataset. Our evaluation shows that Seqping was able to generate better gene predictions compared to three HMM-based programs (MAKER2, GlimmerHMM and AUGUSTUS) using their respective available HMMs. Seqping had the highest accuracy in rice (0.5648 for CDS, 0.4468 for exon, and 0.6695 nucleotide structure) and *A. thaliana* (0.5808 for CDS, 0.5955 for exon, and 0.8839 nucleotide structure).

**Conclusions:**

Seqping provides researchers a seamless pipeline to train species-specific HMMs and predict genes in newly sequenced or less-studied genomes. We conclude that the Seqping pipeline predictions are more accurate than gene predictions using the other three approaches with the default or available HMMs.

## Background

Rapid and cost-effective next-generation sequencing (NGS) technologies produce large volumes of DNA sequencing data in large-scale genome projects. These advances enabled the research community to sequence many plant genomes and transcriptomes. After the assembly process, the next critical step is annotation of these newly sequenced genomes. Experimental methods for gene validation, biological interpretation and annotation are costly, time-consuming, and labor intensive. Hence, there is a pressing need to develop accurate and fast tools to analyze genomic sequences, especially to identify genes and determine their functions. Many computational tools had been developed with intent to solve the gene finding problem. Protein coding genes are commonly predicted using Hidden Markov Model (HMM) approach [1–5], Conditional Random Field [6], Support Vector Machine [7], Neural Network [8, 9], or by combining multiple predictions from various programs [10, 11]. However, gene finders are often trained using known gene models and this leads to biases in gene structure [12–14]. None of these systems incorporates a flexible, universal gene model that can perform gene prediction for a wide range of species. The process is more complex for plants due to its typically large genome size, short exons bordered by large introns, highly repetitive sequences, and alternative spliced transcripts. Currently available gene finders do not accurately predict most of the protein-coding regions [15], and predicting the complete set of an organism’s protein-coding genes remains a significant challenge.

Recently developed automatic pipeline, such as SnowyOwl [16] and CodingQuarry [17] is designed and optimized for fungal genomes, while BRAKER1 [18] is generally for eukaryotic genomes. The main goal of our work was to develop a versatile gene prediction pipeline that could be applied to any newly (even partially) sequenced plant genome. In order to address these issues, we combined existing gene-finders with self-trained HMMs constructed from a training set of the same species to predict gene models. Our program automates and streamlines the gene prediction process by preparing the training dataset, building species-specific HMMs, predicting gene models and compiles the relevant information for the gene models.

## Methods

The scripts run on Linux platform in Bash shell and require some preinstalled software like BLAST+ 2.2.30 [19], CD-HIT 4.5.4 [20], Splign 1.39.8 [21], GlimmerHMM 3.0 [5, 22], AUGUSTUS 2.6.1 [23], SNAP [4], MAKER 2.10 [24, 25], and EMBOSS 6.4.0 [26].

### Scripting

**Fig. 1:**
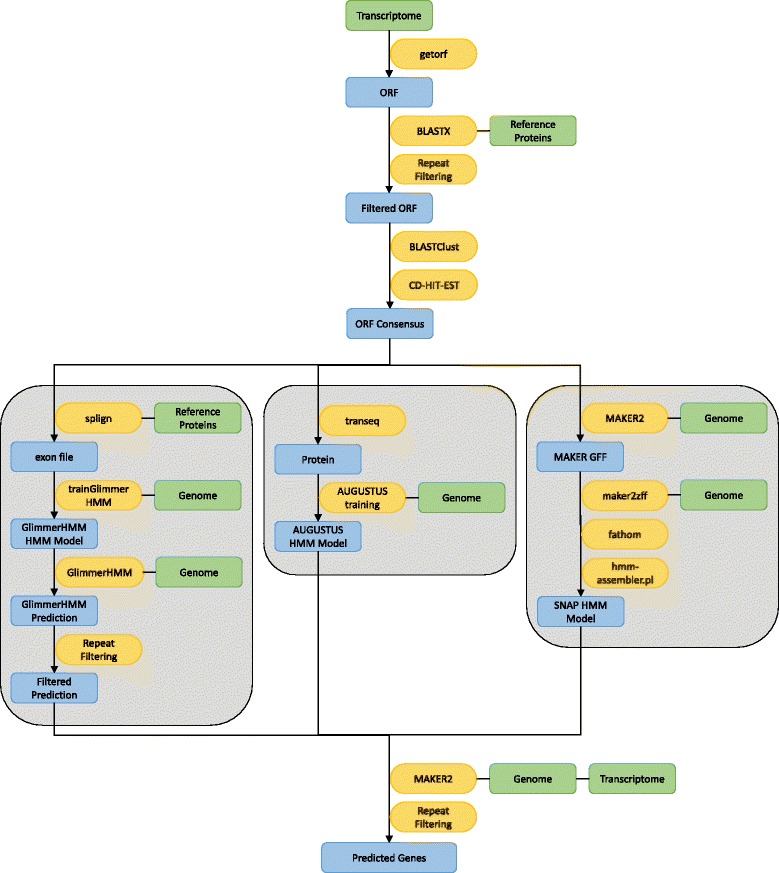
The Seqping self-training gene prediction pipeline. Green boxes indicate input sequences in FASTA format, yellow boxes indicate software or processes, and blue boxes indicate intermediate output files

UNIX based Bash and Perl scripting was used in the current work. “*seqping.sh*” is the main script that executes a sequence of commands, including invoking other scripts written in Bash and Perl. The pipeline is shown in Fig. 1. We divided the task into seven stages: (1) setting up the working directories, (2) preparation of the training set, (3) GlimmerHMM [5, 22] training, (4) AUGUSTUS [23] training, (5) SNAP [4] training, (6) MAKER2 [24, 25] prediction, and (7) results filtering. Seqping supports multiple processors analysis, as well as job submission to Sun Grid Engine (SGE) or Portable Batch System (PBS) job schedulers. The script’s optimized parameters provide an automated and efficient tool for filtering and structural annotation of gene predictions.

### Program input

The user is prompt to submit the respective species’ (1) transcriptome and (2) genome sequence in FASTA format. A reference protein file containing full length protein sequences selected from the NCBI Protein Database [27] is required for validation and annotation of the gene predictions. We selected only proteins from the phylum Magnoliophyta (flowering plants) and excluded hypothetical, ribosomal, mitochondrial and chloroplast proteins. TIGR Plant Repeat [28] and RepBase [29] sequences were combined into a database for TBLASTX filtering while HMM profiles from Gypsy Database [30] were used for HMMER [31] hmmsearch filtering.

### Preparation of training dataset

Transcriptome from the organism of interest is used to generate the training set. Seqping extracts open reading frames (ORFs), sized between 500 and 5000 nucleotides, from the transcriptome using *getorf* tool from the EMBOSS package [26]. Next, ORFs with reference proteins support (BLASTX E-value < E-10) are clustered using BLASTClust and CD-HIT-EST [20] tools with stringent parameters. Transcripts that have similarity to repeats are removed (TBLASTX against TIGR Plant Repeat [28] and RepBase [29] with E-value < 1E-10, and *hmmsearch* against Gypsy Database [30] with E-value < 1E-5). The remaining sequences are used as the training set to develop species-specific HMMs for gene prediction.

In the next step, the program aligns the training set to the genome using Splign and Compart tools [21]. The aligned training set and corresponding genomic sequences are used to train GlimmerHMM [5, 22]. Then a custom Perl script is used to convert the Splign output into an exon file, and *trainGlimmerHMM* is activated to generate a HMM model. Gene prediction by GlimmerHMM is executed using the newly generated species-specific HMM, followed by filtering of repeats. To generate HMM for AUGUSTUS [23], the training set is translated into protein sequences using EMBOSS’s *transeq*. A different HMM is produced using the AUGUSTUS-specific training script that can be found in the AUGUSTUS package. In order to build the HMM for SNAP [4], Seqping runs a basic MAKER2 [24] prediction using DNA and protein sequences from the training set. The SNAP HMM model is finally produced by *fathom* and *hmm-assembler* scripts from the SNAP package.

### Program output

**Fig. 2:**
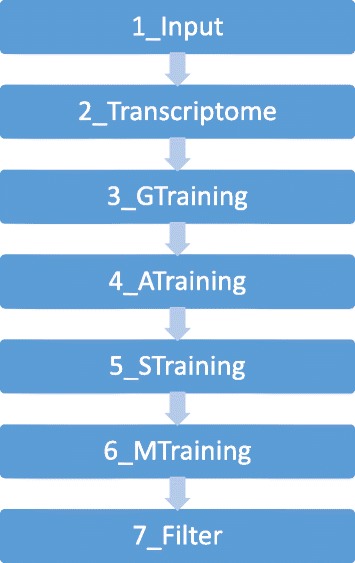
The list of output directories from the seven stages in Seqping

The output is stored in a user-defined directory. The self-trained HMM models and gene prediction outputs are located in several sub-directories labeled by the names of the respective gene-finding modules. MAKER2, which is the final tool to combine all models (GlimmerHMM’s prediction, AUGUSTUS’s HMM and SNAP’s HMM) and evidences (transcriptome data and NCBI Protein Database), generate the list of predicted genes in GFF format, as well as predicted genes and proteins sequences in FASTA format. The list of output directories in a tree-like format is shown in Fig. 2. A comprehensive log file is generated as the pipeline is executed.

### Comparison of gene prediction tools

To demonstrate the effectiveness of the Seqping pipeline, the rice (*Oryza sativa ssp. japonica*) and *Arabidopsis thaliana* genomes were used. Benchmarking Universal Single-Copy Orthologs (BUSCO) [32] analysis of the predicted genes were tested using the 956 plantae BUSCO profiles. The predicted gene models were also compared to manually curated gene sets for both organisms. A total of 24,229 complete genes of *O. sativa* ssp*. japonica* from RefSeq were used as the reference set to calculate sensitivity (Sn) and specificity (Sp). For *A. thaliana,* annotations from TAIR10 [33] was used to compare the performance of Seqping. Sn and Sp were calculated as described by Burset and Guigo [34] using GenomeTools [35] *gt-eval*. Sn and Sp are defined as $\mathrm{S}\mathrm{n}=\frac{\mathrm{TP}}{\ \mathrm{T}\mathrm{P} + \mathrm{F}\mathrm{N}\ }$ and $\mathrm{S}\mathrm{p}=\frac{\mathrm{TP}}{\ \mathrm{T}\mathrm{P} + \mathrm{F}\mathrm{P}\ }$ where TP, FN and FP is the number of true positives, false negatives, and false positives, respectively. Accuracy (Acc) is defined as the average of Sn and Sp, $\mathrm{A}\mathrm{c}\mathrm{c}=\frac{\mathrm{Sn}+\mathrm{S}\mathrm{p}}{\ 2\ }$ [34]. Comparison was done at CDS, exon and single-nucleotide levels.

### Materials

Twelve rice chromosomes were obtained from the MSU Rice Genome Annotation Project release 7 [36]. The transcriptome set contained assembled transcripts from three RNA-Seq projects in NCBI BioProject: PRJNA79825, PRJDA67119, and PRJNA80103. A total of 175,251 assembled transcripts were used as input for the pipeline. The contigs N50 and mean length are 1693 and 956 respectively. For *A. thaliana* gene prediction, the genome and transcriptome data were downloaded from TAIR10 [33] genome release (1,476,275 ESTs and 77,415 cDNAs).

## Results and discussion

Transcriptome data is a key source of experimental evidence for genome annotation, since it reflects the genes that are expressed in specific cell types or conditions [37]. Mapping of a large number of full-length transcripts greatly improves identification of the exon structures of eukaryotic genes [38–40]. It also allows identification of alternative splicing [40, 41], and accurate prediction of transcription start sites and promoters [42, 43]. Incorporation of transcriptome data into the gene prediction pipeline is a feasible and cost-effective approach for annotation of newly sequenced or less-studied genomes [17, 40, 44], in spite of existing computational challenges and complexity of higher eukaryotic genomes [45].

HMMs form the basis for most currently used gene finders. HMMs for gene prediction contain probabilistic state models for different functional parts of the genomic sequence, such as translational and splicing signals and coding regions, depending on the base frequency. The three main gene finders: GlimmerHMM, AUGUSTUS, and SNAP, have pre-build HMM models for several model species in their software packages, but the available existing HMMs may not be suitable for highly complex plant genomes. The prior probabilities calculated for HMM in other species difficultly identify the genes in targeted plant genome. Species-specific HMMs are required to find both novel and well-characterized genes. Seqping performs species-specific HMM training for three programs: GlimmerHMM, AUGUSTUS and SNAP, and uses MAKER2 to combine the predictions in order to take advantages of the different algorithms used by the respective programs. MAKER2 uses the GFF3 file of GlimmerHMM, AUGUSTUS HMM, and SNAP HMM, in addition with the transcriptome data, to generate the final set of predicted genes. All the transcriptome and gene models available are also used by MAKER2 to generate a quality metric called Annotation Edit Distance (AED) for each gene model, in which AED score of 0 is the best-supported gene models.

Seqping also filter repetitive sequences, since these sequences are mainly represented by noncoding sequences. In plants, repetitive sequences may account for up to 90% of the genome [46]. These repeats may also create challenges during the automatic gene finding process. Filtering of repetitive sequences is implemented in several stages in the pipeline, namely during the selection of ORFs for the training set, GlimmerHMM gene prediction and MAKER2 gene prediction. The presence of repetitive sequences is identified by comparison to the TIGR Plant Repeat [28], RepBase [29], and Gypsy Database [30].

### *Oryza sativa* gene prediction

The pipeline was first tested in rice (*O. sativa* ssp*. japonica*). It took ~100 h to execute the gene prediction pipeline on the Linux SGE cluster with 9 nodes (8 CPUs per node). The rice transcripts were treated as described in the Training Set Preparation section, producing 11,729 putative full-length ORFs that were then used for HMM training. The Seqping pipeline, using the new HMMs and transcriptome data identified 24,009 highly confidence rice genes. BUSCO analysis, which is a benchmarking tool to determine the completeness of genome assemblies and annotations, revealed that Seqping was able to identify 95.92% of the highly conserved plant genes (Table 1). This was the best performance, followed by MAKER2 (92.26%), GlimmerHMM (91.53%) and Augustus (88.70%).

**Table 1:** Accuracy of four methods of gene prediction using the *O. sativa* genome

		Seqping^a^	MAKER2^b^	GlimmerHMM^c^	AUGUSTUS^d, e^
BUSCO		95.92%	92.26%	91.53%	88.70%
CDS structure	Sn	0.6175	0.5193	0.4394	0.4717
	Sp	0.5120	0.4922	0.2774	0.3008
	Acc	0.5648	0.5058	0.3584	0.3863
Exon structure	Sn	0.4820	0.4028	0.3089	-
	Sp	0.4116	0.3880	0.2129	-
	Acc	0.4468	0.3954	0.2609	-
Nucleotide Level	Sn	0.6906	0.5950	0.6597	0.6581
	Sp	0.6484	0.6680	0.4381	0.3698
	Acc	0.6695	0.6315	0.5489	0.5140

^a^Seqping: Trained using rice transcriptome; ^b^MAKER2: SNAP’s rice HMM, AUGUSTUS’s maize model and rice transcriptome; ^c^GlimmerHMM: Trained using rice transcriptome; ^d^AUGUSTUS: maize model

^e^Using the available maize models, AUGUSTUS does not predict exon structure

It also had the highest Sn, Sp and Acc for three comparison levels (CDS, exon, single-nucleotide), with the exception of the Sp at the nucleotide level, in which it scored the second highest score of 0.6484 after MAKER2 (0.6680). This shows that the Seqping pipeline was able to produce the most precise rice models compared to MAKER2, GlimmerHMM and AUGUSTUS. It also indicates that optimization of parameters to train the gene finders in Seqping was an important step to enable the gene prediction software to accurately identify gene structure. The predicted rice genes from Seqping were also independently verified using a different approach. Comparison of the Seqping models to the MSU annotation using ParsEval [47] yielded 87.70% shared gene loci. These results indicate that Seqping had the best prediction for the rice genome.

### *Arabidopsis thaliana* gene prediction

Using the Seqping pipeline, a total of 25,829 putative full-length ORFs were identified and used for the HMM training. The pipeline identified 21,229 highly confidence genes. BUSCO analysis showed that AUGUSTUS was able to identify the highest number (98.64%) of conserved orthologs. This was followed by GlimmerHMM (98.12%). Seqping was ranked as the third, with 96.44% identified. Nevertheless, it was also still able to identify more than 95% of the orthologs available.

To compare the performance of the four programs, TAIR10 [33] *A. thaliana* annotations were used as the reference gene set (Table 2). Overall, the Sn for CDS structure was much lower compared to rice as the annotations from TAIR10 covers many alternative splicing forms. Seqping had the best Sn at the exon level and Sp at the nucleotide level, while MAKER2 performed better in Sp at the CDS and exon levels. GlimmerHMM achieved the highest Sn for nucleotide structure. Augustus was able to predict the best Sn at CDS structure. Nevertheless, Seqping had the best overall Acc at all three levels. This shows that while each tool was sacrificing either Sn or Sp, Seqping was able to balance the predictions by using a combination of the tools.

**Table 2:** Accuracy of four methods of gene prediction using the *A. thaliana* genome

		Seqping^a^	MAKER2^b^	GlimmerHMM^c, e^	AUGUSTUS^d^
BUSCO		96.44%	94.14%	98.12%	98.64%
CDS Structure	Sn	0.2749	0.0738	0.2804	0.3075
	Sp	0.8867	0.8877	0.7515	0.7527
	Acc	0.5808	0.4808	0.5160	0.5301
Exon structure	Sn	0.4596	0.1207	-	0.4155
	Sp	0.7313	0.7457	-	0.5373
	Acc	0.5955	0.4332	-	0.4764
Nucleotide Level	Sn	0.7929	0.1932	0.9350	0.9634
	Sp	0.9748	0.9750	0.8150	0.7974
	Acc	0.8839	0.5841	0.8750	0.8804

^a^Seqping: Trained using *A. thaliana* transcriptome; ^b^MAKER2: SNAP’s *A. thaliana* HMM, AUGUSTUS’s *A. thaliana* model and *A. thaliana* transcriptome; ^c^GlimmerHMM: *A. thaliana* model; ^d^AUGUSTUS: *A. thaliana* model

^e^Using the available *A. thaliana* model, GlimmerHMM does not predict exon structure

## Conclusions

The Seqping pipeline predictions are more accurate compared to the other three approaches with the default or available HMMs. We demonstrated that integration of multiple tools result in higher quality gene predictions in both dicotyledon and monocotyledon plants. By training species-specific HMMs, Seqping provides an effective, organism independent, gene prediction tool for non-model plant species. Expectedly, the performance is influenced by the quality of the transcriptome and genome sequences of the target species. The pipeline is most suitable for used in newly sequenced or less-studied plant genomes.
